# A Transient Hermaphroditic Stage in Early Male Gonadal Development in Little Yellow Croaker, *Larimichthys polyactis*

**DOI:** 10.3389/fendo.2020.542942

**Published:** 2021-01-27

**Authors:** Qing-Ping Xie, Bing-Bing Li, Wei Zhan, Feng Liu, Peng Tan, Xu Wang, Bao Lou

**Affiliations:** ^1^ Institute of Hydrobiology, Zhejiang Academy of Agricultural Sciences, Hangzhou, China; ^2^ Marine Fisheries Research Institute of Zhejiang Province, Zhoushan, China; ^3^ School of Fishery, Zhejiang Ocean University, Zhoushan, China; ^4^ Department of Pathobiology, College of Veterinary Medicine, Auburn University, Auburn, AL, United States; ^5^ Alabama Agricultural Experiment Station, Auburn, AL, United States; ^6^ The HudsonAlpha Institute for Biotechnology, Huntsville, AL, United States

**Keywords:** hermaphrodite, sex differentiation, hermaphroditism, gonochorism, intersex gonadal stage

## Abstract

Animal taxa show remarkable variability in sexual reproduction, where separate sexes, or gonochorism, is thought to have evolved from hermaphroditism for most cases. Hermaphroditism accounts for 5% in animals, and sequential hermaphroditism has been found in teleost. In this study, we characterized a novel form of the transient hermaphroditic stage in little yellow croaker (*Larimichthys polyactis*) during early gonadal development. The ovary and testis were indistinguishable from 7 to 40 days post-hatching (dph). Morphological and histological examinations revealed an intersex stage of male gonads between 43 and 80 dph, which consist of germ cells, somatic cells, efferent duct, and early primary oocytes (EPOs). These EPOs in testis degenerate completely by 90 dph through apoptosis yet can be rescued by exogenous 17-*β*-estradiol. Male germ cells enter the mitotic flourishing stage before meiosis is initiated at 180 dph, and they undergo normal spermatogenesis to produce functional sperms. This transient hermaphroditic stage is male-specific, and the ovary development appears to be normal in females. This developmental pattern is not found in the sister species *Larimichthys crocea* or any other closely related species. Further examinations of serum hormone levels indicate that the absence of 11-ketotestosterone and elevated levels of 17-*β*-estradiol delineate the male intersex gonad stage, providing mechanistic insights on this unique phenomenon. Our research is the first report on male-specific transient hermaphroditism and will advance the current understanding of fish reproductive biology. This unique gonadal development pattern can serve as a useful model for studying the evolutionary relationship between hermaphroditism and gonochorism, as well as teleost sex determination and differentiation strategies.

## Introduction

Hermaphroditism and gonochorism are the most important sex determination systems and sex differentiation methods in sexually reproductive organisms. Hermaphroditism rarely occurs in tetrapods, amphibians, reptiles, birds, or mammals, while it occurs commonly in fish, invertebrates, and plants (1–5). The majority of teleost fish are gonochoristic, including large yellow croaker (*Larimichthys crocea*), tilapia (*Oreochromis niloticus*), medaka (*Oryzias latipes*), and many others (6–9). Nevertheless, hermaphroditism has been documented in more than 1,500 species (10).

In fish, hermaphroditism can be classified into three types, sequential hermaphrodite’s protandry, sequential hermaphrodite’s protogyny, and simultaneous hermaphrodites, which is quite rare (11). During the development of protandrous fish species, the testis matures first and produces sperm. After sperm excretion, the male reproductive organs degenerate, and then the ovaries gradually mature and form functional ovaries. These species include black porgy (*Acanthopagrus schlegeli*), sea bass (*Lates calcarifer*), gilthead seabream (*Sparus aurata*), and anemonefish (*Acanthopagrus schlegeli*) (12–16). In contrast, the ovary is the first developed gonad in protogynous species, then some or all individuals undergo sex change to form functional testis. These species include ricefield eel (*Monopterus albus*), groupers (*Epinephelus coioides*, *Epinephelus akaara*, and *Epinephelus awoara* et al.), mudskipper (*Pseudapocryptes lanceolatus*), and ballan wrasse (*Labrus bergylta*) (17–22). In addition, simultaneous hermaphroditic fish have both testicular and ovarian tissues present in a single individual at the same time. This type of fish is relatively rare, and the most representative species is the mangrove killifish (*Kryptolebias marmoratus*), which can even reproduce by self-fertilizing (23, 24). At last, zebrafish represents a special mode of gonadal development and differentiation. As a gonochoristic species, immature ovarian tissues first develop in both sexes, a stage known as hermaphrodite ovarian stages or juvenile hermaphrodite. After that, the juvenile ovaries differentiate further into true ovaries without tissue remodeling in genetic females. For genetic males, the differentiation of testis involves a process of degeneration (apoptosis) of the oocyte-like germ cells, which is accompanied by stromal tissue remodeling for differentiation into testicular tissues (5, 25, 26).

Estrogen is a critical factor for sex determination and gonadal differentiation in teleosts. It is well documented that estrogens and their main synthase (aromatase) encoded by *cyp19a1a* (Cytochrome P450 Family 19 Subfamily A Member 1) play a vital role in promoting ovarian development and maintaining feminization afterward in both hermaphroditic or gonochoristic teleosts (12, 27–29). Loss of function of *cyp19a1a* by CRISPR/Cas9 and TALENs will lead to complete masculinization in zebrafish and sexual reversion from female to male in XX tilapia, respectively (5, 30). Long-term treatment using Fadrozole, an estrogen-synthase aromatase inhibitor, can turn a differentiated ovary into a functional testis, and this secondary sex reversal could be rescued by estrogen (31). In contrast, juvenile fish exposed to estrogens will result in complete sex reversal in genetic males of gonochoristic species or transient sex reversal in protandrous species (32–35). Simultaneous exposure to estrogen and androgen also resulted in feminization in tilapia (36). Thus, research on estrogen levels and expression regulation during the sexual transitional period will help understand sex differentiation strategies in both hermaphroditic and gonochoristic teleost.

Little yellow croaker (*Larimichthys polyactis*, Bleeker, 1877), also named the small yellow croaker or yellow corvina, is a croaker native to the western Pacific. It is one of the most famous fish species of great economic importance in China, Korea, and Japan. A few decades ago, the abundance of wild *L. polyactis* had severely declined since the 1980s, and its commercial value has increased significantly due to overfishing, seawater pollution, as well as ocean current and water quality changes (37, 38). Although the resources of *L. polyactis* had been gradually recovered ever since (39), overfishing caused sexual precocity, gender imbalance, and growing miniaturization in *L. polyactis* populations (40, 41). Therefore, artificial breeding techniques are in urgent need of restoring fishery resources. Characterizing the sex determination/differentiation and gonadal development process will significantly advance knowledge in its reproductive physiology, which is currently lacking. In 2015, an artificial breeding program was successfully established by artificial spawning of wild *L. polyactis* and larvae culture (42, 43). In this study, samples of an artificial breeding population of *L. polyactis* were obtained from generation F3 (2017) to F6 (2020) to investigate gonadal differentiation. The process of gonadal development was analyzed by histological and endocrinological approaches. We have established an excellent model for studying the relationship of hermaphrodite and gonochorism in the teleost, and the discovery of a transient juvenile intersex stage in males also enriches the theory of teleost sex determination and differentiation.

## Materials and Methods

### Animals

Artificial breeding of little yellow croaker (*Larimichthys polyactis*) F3-F6 generation individuals were obtained from the Xiangshan harbor aquatic seedling co. LTD (Xiangshan County, Ningbo, China) and the Marine Fishery Institute of Zhejiang Province (Xixuan Island, Zhoushan, China). *L. polyactis* broodstock was kept in running aerated seawater in cement ponds with a smooth surface at natural water temperature under a natural photoperiod. Larvae were fed with rotifers (*Brachionus sp*) initially, and microalgae (*Chlorella sp*) from 4 to 15 days post-hatching (dph). From 12 to 30 dph, the larvae were fed with newly hatched *Artemia nauplii* (Aquamaster, Binzhou, China). Juvenile fish were fed on a standard commercial diet since 20 dph. After two months, the juveniles were transferred into 20 m^3^ ponds at a density of ~200 individuals per m^3^ for field grow-out and were reared in natural seawater at 20.0 to 27.0°C temperature. The fish fry was fed twice daily with commercial feed. Animal experiments were conducted in accordance with the regulations of the Guide for Care and Use of Laboratory Animals, which was approved by the Committee of Laboratory Animal Experimentation at Zhejiang Academy of Agricultural Sciences.

### Gonadal Morphology, H&E Staining Histology and Sex Ratio Statistics

For morphology analyses, the ovary and the testis were dissected at 90 and 360 dph. For the early stages (before 90 dph), the abdominal cavity was dripped with the Bouin’s fixation until the gonads were completely covered and turned yellow, and excessive fluid was removed. For histology analyses, the ovary and testis were dissected at 7, 20, 30, 40, 43, 46, 49, 50, 60, 70, 80, 90, 100, 120, 180, 240 and 270 dph. For post-larvae and small juveniles (7–40 dph) in which gonads were too small to be separated, all tissues from the body cavity were included for analysis. For large juveniles and post juveniles, both gonad lobes (40–240 dph) or the large lobes (270 dph) were collected and cut into small pieces (10 mm) for better fixation in Bouin’s solution on a shaker for 24 h at room temperature. After fixation, the tissues were dehydrated through a series of ethanol concentration gradients, embedded in paraffin, sectioned at 5 μm, and stained with hematoxylin and eosin. An Axio Imager 2 light microscope (Carl Zeiss Microscopy GmbH, Jena, Germany) was used to image the stained sections. The sex ratio statistics from 43 to 270 dph were based on histological observation.

### Germ Cell Count

The number of germ cells was counted in 6 complete sections after H&E staining. Microscopic examinations of each sex were performed at 40, 50, 60, 70, and 80 dph.

### TUNEL Assay

Terminal deoxynucleotidyl transferase (TdT)-mediated dUTP-digoxigenin nick-end labeling technique was applied to evaluate the apoptotic response of EPOs in the testicular hermaphroditic stage. Reactions were performed on sections (5 µm) of 4% Paraformaldehyde (PFA)-fixed (overnight at 4°C) and paraffin-embedded *L. polyactis*’ ovaries and testes at 80 dph. Apoptotic cells with DNA breaks were detected using Colorimetric TUNEL Apoptosis Assay Kits (#C1091, Beyotime Institute of Biotechnology, Shanghai, China) and In Situ Cell Death Detection Kit, TMR red (#12156792910, Roche, Indianapolis, IN, USA). TUNEL assay was then performed according to the instructions by the manufacturer. For TMR red kit, DNA was stained with DAPI. Images were captured using a Zeiss light microscope Axio Imager 2 and Zeiss confocal microscope LSM710. Image processing and analysis were performed using AxioObserver (Carl Zeiss Microscopy GmbH, Jena, Germany) and ZEN software.

### Administration of Exogenous E2

Fish in three parallel treatment and control groups were kept in 1-ton tanks with dark blue and smooth material walls, at a density of 150 fish per tank/group. The treatment groups were directly exposed to E2 for 2 h each day. E2 (17*β*-estradiol, CAS: 50-28-2, ≥98%) purchased from Sigma (#E8875, Sigma-Aldrich, Shanghai, China) was dissolved in 95% ethanol as a 4 mg/ml stock solution, which was stored at 4°C. The final concentration of E2 in the treatment water was 10 μg/L. The treatment was applied from 80 to 120 dph, and the fish gonads were randomly sampled (N = 30) at 120 dph for statistical and histological analyses.

### Measurement of Steroid Hormones

Blood samples were collected from the caudal veins at 50, 60, 70, 80, 90, 100, 120, 180, 240, 270, 340, 360, 540, and 720 dph, and kept at 4°C overnight. Serum samples were collected after centrifugation at 3,000 rpm for 10 min and stored at −20°C until use. For large juveniles and post-juvenile stages (50–180 dph and after 180 dph), matching blood samples were collected from both sexes, and sex-typing was performed based on histological results. Serum E2, 11-KT, and testosterone levels were measured using the Enzyme Immunoassay (EIA) Kit (Cayman, USA) following the manufacturer’s protocols (N = 6 for each sex).

### Statistical Analysis

Total length, body length, and body weight were measured in 20 randomly sampled male and female fishes at 360 and 720 dph. Thereafter, a gonadosomatic index (GSI = gonad weight/body weight × 100) was calculated. Sex ratios of *L. polyactis* were quantified based on histology results at 43, 46, 49, 50, 60, 70, 80, 90, 100, 120, 180, 240, and 270 dph. The mean number of germ cells and the variance were calculated. Serum E2, 11-KT, and testosterone levels uses two-way ANOVA with sex and time as independent variables. Data for each sex or time were analyzed using software GraphPad Prism 8.0 (San Diego, USA). Statistical software uses SPSS statistics v17.0 (Chicago, USA). Independent sample t-test, one-way ANOVA, and Duncan’s *post hoc* test were performed to determine the statistical significance at *P* < 0.05.

## Results

### A Transient Hermaphroditic Stage Occurs in Male Gonadal Development

To examine the gonadal differentiation process in *L. polyactis*, gonad time series samples were collected and investigated using morphological and histological approaches.

**Figure 1:**
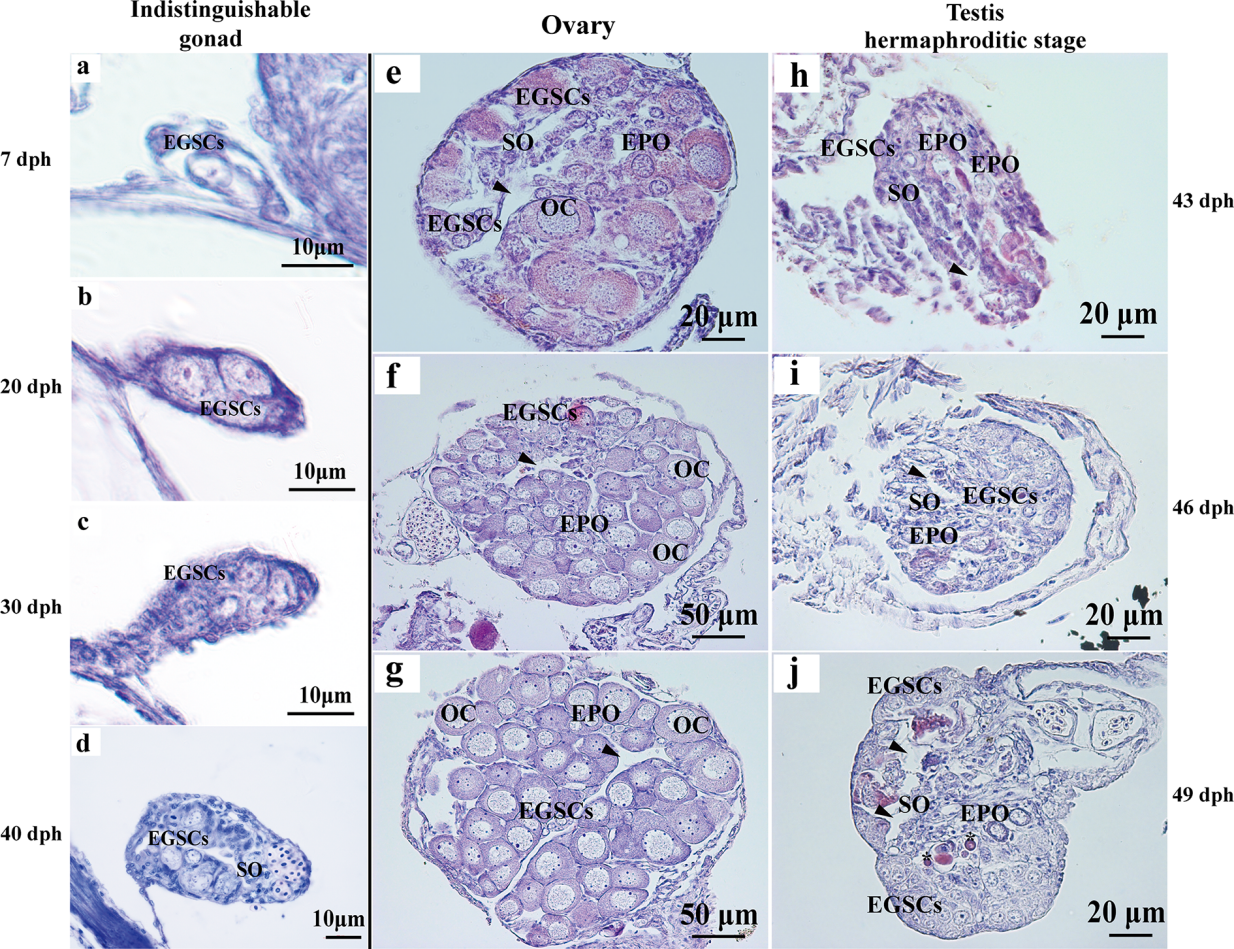
Histology of *L. polyactis* gonads before, during, and after sex differentiation. Histologically, reproductive primordium and primordial germ cell (PGC) formed at 7 dph **(A)**. Germ cells and somatic cells were visible from 7 to 40 dph, and ovary and testis cannot be distinguished at these developmental stages **(A–D)**. The ovary initiates differentiation and development from 43 dph **(E–G)**, whereas the testis initiate differentiation and development and testicular EPOs was observed as early as 43 dph **(H)**. the obvious sperm duct appeared at 46 dph **(I)**, EPOs degeneration was observed from 49 dph, and EPOs degeneration formed apoptotic body was also observed at 49 dph **(J)**. Arrowhead represents the ovary cavity and sperm duct in ovary and testis, respectively. *represents apoptotic bodies. EGSCs, embryonic germ stem cells; SO, somatic cell; OC, oocytes; EPOs, early primary oocytes.

Histologically, the reproductive primordium is formed, and embryonic germ stem cells (EGSCs) are produced at 7 dph (**Figure 1A**). During the gonadal development from 7 to 40 dph, the number of EGSCs is increasing, but the ovary and the testis are histologically indistinguishable at these early stages (**Figures 1A–D**). Meiosis in the ovary is initiated around 43 dph. Early primary oocytes (EPOs) start to develop, and oocytes begin to form at this stage, in which the first meiotic division proceeds to the diplotene stage. The somatic cells clusters for the ovarian cavity formation are also visible (**Figure 1E**). After 40 dpf, the ovarian development progresses rapidly, which is manifested as the expansion of ovary size and an increasing number of oocytes in the gonad (**Figures 1F, G** **)**. Compared with the ovary, fewer EGSCs and more somatic cells are present and in the testis at 43 dpf. Surprisingly, we also observed scattered EPOs occurring in the testis when the sperm duct is visible between 43 and 49 dpf (**Figures 1H–J**). The testicular development is significantly slower than the ovary after EPOs start to degenerate through apoptosis in 49 dpf testis, where apoptotic bodies are observed (**Figure 1J**). The number of germ cells increases gradually during this transitioning stage (43–39 dpf, **Figures 1H–J**).

**Figure 2:**
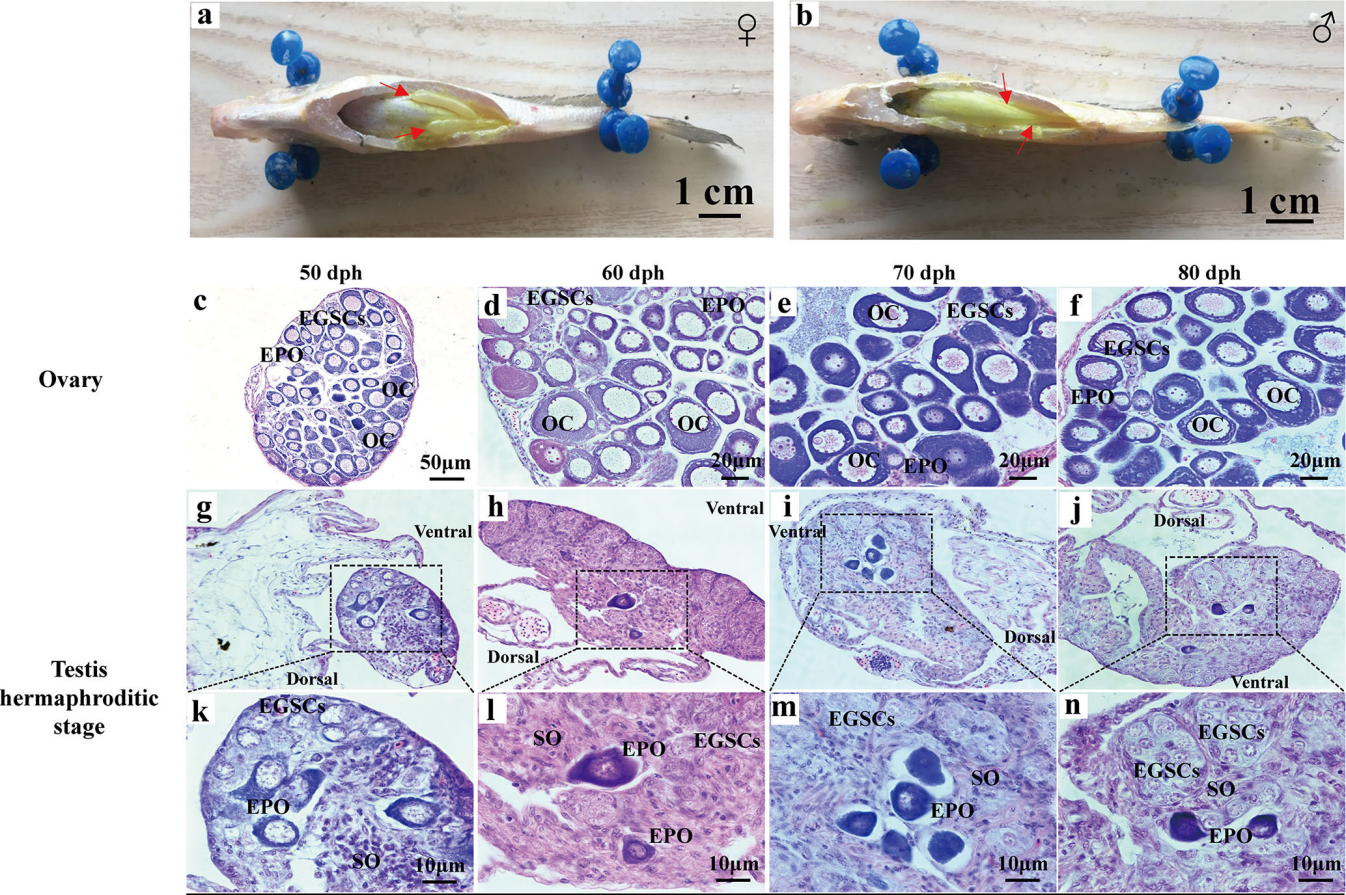
Early gonadal development in male and female juvenile *L. polyactis*. Anatomical and histological examination of the gonads in the juvenile fish. The gonads were indicated by a red arrow **(A, B)**. Early ovary and testis developmental stages were shown in **(C–F)** and **(G–N)**, respectively. EGSCs, embryonic germ stem cells; SO, somatic cell; OC, oocytes; EPOs, early primary oocytes.

At 90 dph, a pair of cigar-shaped ovaries is observed, one of which is slightly longer (**Figure 2A**). The male testes are longer and thinner than ovaries (**Figure 2B**). The number of EPOs increases as they continue to develop to oocytes at 50–80 dph (**Figures 2C–F**). At 50 dph, EPOs frequently occur near the efferent duct and on the dorsal side (**Figure 2G**), suggesting an intersex gonad with the ability to produce both male and female germ cell precursors. However, the testis EPOs’ development was stagnated, and they start to degenerate from 49 to 80 dph (**Figures 1I–J** **;** **Figures 2G–N**). Distinct germ cell cyst formation occurs from ventral to dorsal in the testis at 60 dph (**Figure 2L**). EPOs were found in 100% histological samples of male gonads at 43**–**80 dph in all four breeding season (N = 111), indicating this male-specific hermaphroditic stage is constitutive, rather than stress-induced variable misregulation of gonad development. Therefore, we define this as the transient hermaphroditic stage in male gonad development.

**Figure 3:**
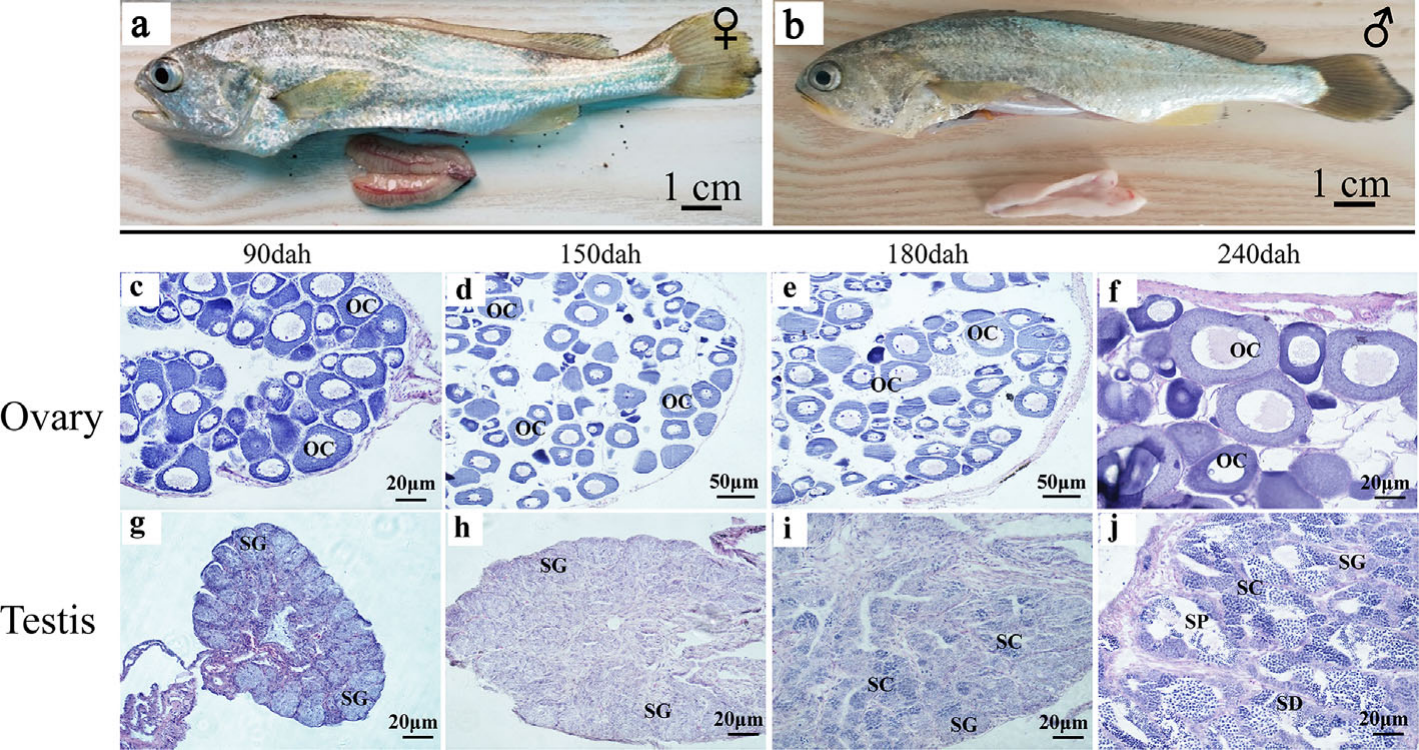
Testis and ovary maturation process in *L. polyactis*. Anatomical and histological examination of the gonads in the adult fish. **(A, B)**. Dissected female **(A)** and male **(B)** gonads at 360 dph. **(C–H)**. Histology images during ovarian development at different time points, showing the oocyte growth in number and size at 90**–**240 dph. **(G–J)**. Histology images showing spermatogonia development and test maturation). OSCs, oogonia stem cell; OC, oocytes; SSCs, spermatogonia stem cell; SC, spermatocytes; SD, spermatid; SP, sperm; SO, somatic cell.

When the gonads mature at 360 dph, the ovaries have fully developed vascular tissues and are filled with full-grown oocytes (**Figure 3A**). The ovary size is not symmetric, and one is longer than the other. The testis consists of two milky-white fan-shaped strips of tissues, one of which is also slightly longer (**Figure 3B**). During ovarian development, oogonial stem cells (OSCs) appear at 90 dph, and oocytes continue to grow in number and size between 90 and 180 dph (**Figures 3C–E**). Phase III oocytes start to appear at 240 dph (**Figure 3F**). In the developing testes, testis oocytes are completely degraded by 90 dph, when the spermatogonial stem cells (SSCs) start to divide through mitosis during 90 to 150 dph (**Figures 3G, H**). Meiosis begins in primary spermatocytes at 180 dph (**Figure 3I**). Secondary spermatocytes, spermatids, and spermatozoa are produced at 240 dph (**Figure 3J**).

### Early Primary Oocytes Degenerate by Apoptosis and Are Rescued by Administration of Exogenous E2

**Figure 4:**
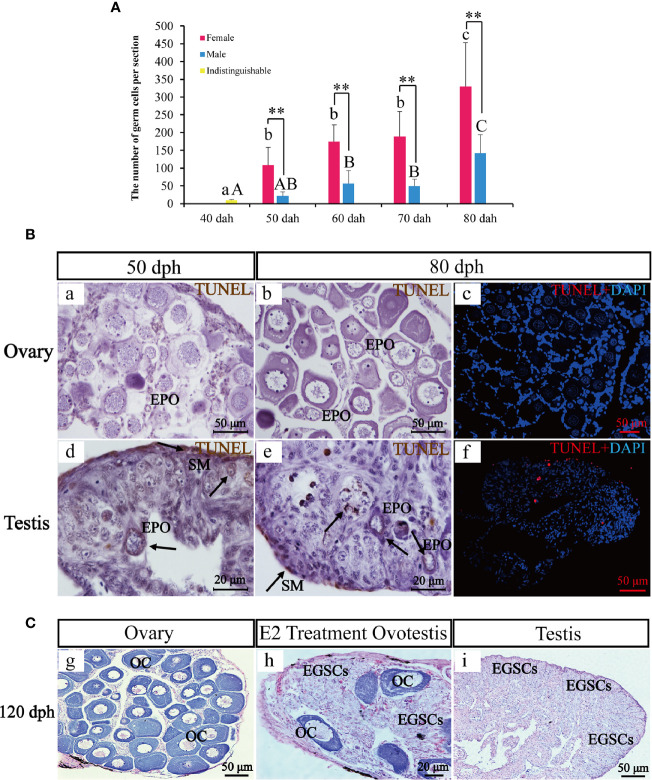
The transient hermaphroditic stage germ cell count, TUNEL assay, and administration of E2. Changes in germ cell numbers from 40 to 80 dph **(A)**. Two TUNEL staining, brown and red fluorescence, represent a positive signal, the blue fluorescence represents DAPI. Positive signals were detected in the perinuclear region of EPOs, part of EGSCs, and somatic cells of the gonad membrane in testes. Few positive signals have been detected in the ovaries **(B)**. Histological examination of the E2 treatment and control fish at 120 dph. EGSCs, embryonic germ stem cells; OC, oocytes **(C)**. Data were presented as means ± SD. ** represent statistically significant levels at P < 0.01 (Independent t-test).

Germ cells numbers were counted in six complete sections for each sex and each timepoint between 40 and 80 dph (**Figure 4A**). Statistical analysis showed that germ cell number at 40 dph was 10 +/− 2.19, when the sex is not distinguishable. Later in development, the germ cell number in the ovary was 2.3**–**5.0 fold higher than testis at 50, 60, 70, and 80 dph (108.5 +/− 50.09 *vs*. 22.0 +/− 11.21, *P*-value = 0.007 at 50 dph; 174.33 +/− 47.41 *vs*. 56.67 +/− 36.94, *P*-value = 0.001 at 60 dph; 189.33 +/− 70.01 *vs*. 48.83 +/− 19.96, *P*-value = 0.001 at 70 dph; 329.67 +/−123.71 *vs*. 142.0 +/− 52.27, *P*-value = 0.007 at 80 dph). The results indicate significant sex differences in germ cell numbers.

To assess potential roles of apoptosis in testis EPO degeneration, we performed TUNEL staining to detect signatures of apoptosis in ovaries and testes at 50 and 80 dph. Positive signals were detected in the perinuclear region of EPOs, in some EGSCs and somatic cells of the gonad membrane in testes only. In contrast, little to no signal of apoptosis were observed in the ovaries (**Figure 4B**), confirming that the presence of apoptosis in EPOs at 50 dph is testis-specific.

An exogenous E2 exposure treatment assay was used to explore estrogen’s involvement in the maintenance and degeneration of EPOs. We observed that 50% of the males developed ovotestis in response to E2 exposure. In these E2-treated ovotestes, oocytes persist with abundant EGSCs (**Figure 4C**). In the remaining males, testicular development was repressed, and the testes were smaller than the control. EPO degeneration was observed based on histological examinations. The results indicated that EPOs are maintained by the administration of exogenous E2, at least in half of the males in our experiments.

### *Larimichthys polyactis* Sex Ratio and Sexual Dimorphism in Body Weight

**Figure 5:**
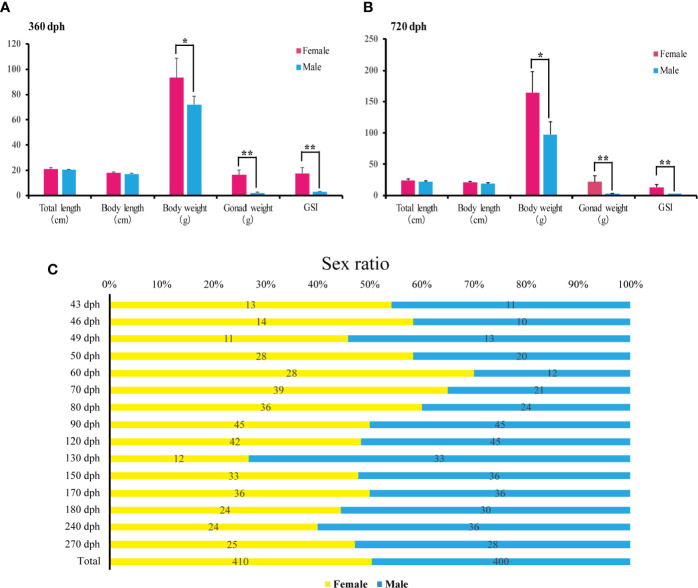
Sex ratios and body weight measurements during *L*. *polyactis* development. **(A, B)**. Barplot of the total length, body length, body weight (W), gonad weight (G), and the gonadosomatic index (GSI = G/W × 100) for females (pink) and males (blue) at 360 dph **(A)** and 720 dph **(B)**. 20 females and 20 males were samples for each timepoint. Data were presented as means ± SD. * and ** represent statistically significant levels at *P* < 0.05 and *P* < 0.01 (Independent t-test). **(C)**. Sex ratios from 43 to 270 dph calculated by relative female and male counts. A total of 410 females and 400 males were sampled.

The female and male’s total length, body length and body weight were measured at 360 and 720 dph (N = 20 for each sex). Statistical analyses showed that females are significantly heavier than males (93.34 +/− 15.51 g *vs*. 71.84 +/− 6.82 g, *P*-value = 0.011 at 360 dph; 164.34 +/− 34.13 g *vs*. 97.49 +/−19.95 g, *P*-value = 0.002, at 720 dph), while no significant differences were found in total length and body length at 360 and 720 dph (*P*-value > 0.05). Additionally, ovary weight was significantly higher than testis (16.29 +/−3.87 g *vs*. 2.00 +/−0.58, *P*-value = 0.000, at 360 dph; 21.98 +/−9.92 g *vs*. 3.03 +/− 0.63, *P*-value = 0.001, at 720 dph), and GSI (gonadosomatic index) of females was significantly higher than males at 360 and 720 dph (17.69 +/− 4.37 g *vs*. 2.76 +/− 0.60, *P*-value = 0.000, at 360 dph; 14.74 +/− 4.39 g*vs*. 3.14 +/− 0.49, *P*-value = 0.006, at 720 dph) (**Figures 5A, B**).

To examine the sex ratios in the artificial breeding *L. polyactis* population, we sampled individuals from 43 to 270 dph over the previous four breeding seasons. The numbers of total female and male are 410 and 400, respectively (**Figure 5C**), suggesting no deviation from a 1:1 sex ratio. Therefore, under the present rearing conditions, the percentage of phenotypic sex was maintained in equal proportions in *L. polyactis*.

### 17*β*-Estradiol, the Primary Female Sex Hormone, Is Elevated During the Male-Specific Transient Hermaphroditic Stage

**Figure 6:**
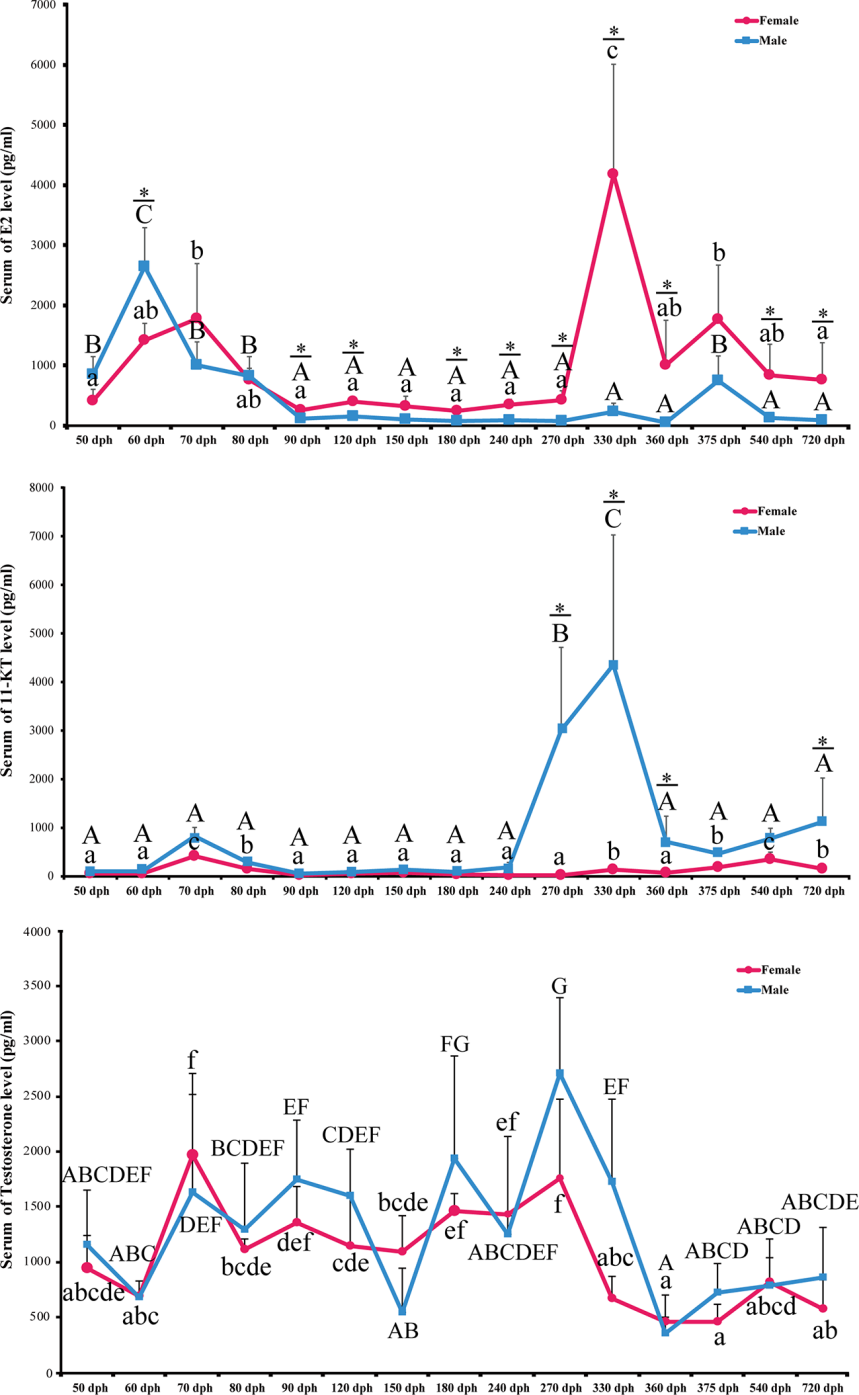
Level of serum E2, 11-KT. and Testosterone during *L*. *polyactis* development. The serum of E2, 11-KT and Testosterone levels in females and males fish was plotted at different development time points. The results were presented as the means ± SD from a sample of six individuals for each data point. Significant analyses were conducted by two-way ANOVA tests with sex and time as independent variables. Different uppercase and lowercase letters above the error bar indicate statistical differences between females and males at different time points, respectively, at *P* < 0.05 as determined by one-way ANOVA followed by Duncan’s *post hoc* test. * represent a significant difference at *P* < 0.05 between females and males, respectively, by independent t-test.

To elucidate the role of endocrine hormones in the hermaphrodite intersex stage, we collected serum samples during the complete spawning cycle at 50, 60, 70, 80, 90, 120, 150, 180, 240, 270, 330, 360, 375, 540, and 720 dph. 17*β*-estradiol (E2), 11-keto-testosterone (11-KT), and testosterone were measured using the Elisa kit (N = 6 for each sex). In females, serum E2 levels were slightly increased in 50 to 70 dph. E2 decreased to the baseline at 90 dph and remained low until 270 dph. A dramatic elevation of E2 levels started at 270 dph and peaked at 330, which is the preoviposition period (**Figure 6**).

The E2 level is low in males except for 50 to 80 dph, which corresponds to the transient hermaphroditic stage (**Figure 6**). Serum E2 levels in male fish increased at 50 dph and peaked at 60 dph, which is significantly higher than in females (2,648.64 +/− 621.80 pg/ml *vs*. 1,424.17 +/− 274.29 pg/ml, *P*-value = 0.022). After 60 dph, the E2 level decreased to a low level and remained low from 80 to 360 dph. Significant interactions between sex and time were detected for E2 concentrations in serum [*P* < 0.0001, two-way ANOVA test].

### 11-Ketotestosterone Is at a Low Level at the Male Transient Hermaphroditic Stage and Peaked During Testis Maturation Between 240 and 360 dph

Serum 11-KT in females remains at a very low level through the entire egg-laying cycle. Male 11-KT levels are also low before 240 dph, and there was no significant difference between males and females. A rapid increase of 11-KT in males was observed at 270 dph, and it reached the highest level at 330 dph. After that, 11-KT decreased at 360 dph and was significantly higher than females until 720 dph (**Figure 6**, *P*-value < 0.05). As expected, the interaction between sex and time was also significant [*P* < 0.0001, two-way ANOVA test].

We also examined the levels of serum testosterone. It fluctuates during the spawning cycle, but there is no significant difference between males and females (*P*-value > 0.05). The sex with time interaction term is not significant either [*P* = 0.15, two-way ANOVA] (**Figure 6**).

## Discussion

### *Larimichthys polyactis*’ Hermaphroditic Stage Is Transient and Male Specific

**Figure 7:**
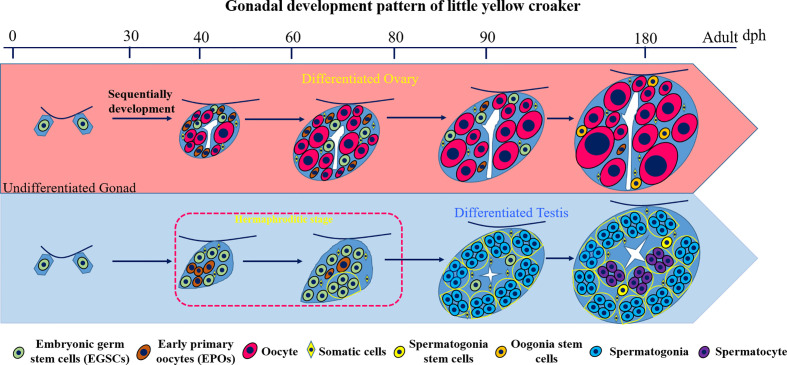
Diagram of the gonad development pattern in *L*. *polyactis*. Dph, days after hatching. Green: EGSCs, embryonic germ stem cells; brown: EPOs, early primary oocytes; Red: oocytes. Yellow diamond: SM, somatic cells; Yellow: SSCs, spermatogonia stem cells; Orange: OSCs, Oogonia stem cells; Blue: spermatogonia. Purple: spermatocyte.

In this study, we investigate the gonad development process in *L. polyactis* and characterized a novel and unique pattern of sex differentiation (**Figure 7**). The gonadal sex is indistinguishable before 40 dph. After 40 dph, the ovary development is continuous with increasing number and size of oocytes toward a functional ovary. In male gonad development, we discovered a 37-days transient hermaphroditic stage, which is characterized by the presence of early primary oocytes (EPOs) in testes. Mechanistically, significant elevation of the 17*β*-estradiol (E2) level and low level of 11-KT were observed in the developing testis. It appears that the testis development started with the production of EPOs, followed by the degeneration of these immature oocytes, and testicular differentiation starts from ventral to dorsal. The EPOs frequently occur near the efferent duct and on the dorsal side. This is similar to what is observed in female-to-male secondary sex reversal, in which testis development starts from ventral to dorsal (29). The testis resumes its normal mitosis and meiosis after this period and gradually develops into a functional testis.

To our knowledge, this pattern has not been reported in any other fish, including closely related species. The *Larimichthys* genus has four species, *Larimichthys crocea* (*L*. *crocea*), *L*. *polyactis*, *L. pamoides*, and *L. terengganui* (44). According to the phylogenetic relationship, the *L. crocea* and the *L. polyactis* are the most closely related sister species (45). *L*. *crocea* is a differentiated gonochorist (46). The formation of ovarian cavity and meiosis of germ cells begin at 60 dph, and primary oocytes are present by 120 dph. The differentiation of testis begins at 95 dph. Meiosis is initiated at 215 dph, and testis lobules start to form at 230 dph. An intersex stage was not observed in the *L. crocea*.

One possible explanation is that the transient hermaphroditic stage is facultative in testis development due to abnormally increased E2 level at 50 dph. If this was true, we would observe variations in the testis developmental pattern, in terms of EPOs presence and the timing of E2 level elevation. However, anatomical and histological results from four consecutive years with at least 120 samples per year showed that all male gonads have EPOs presence and signs of EPOs degeneration at 49−80 dph. Our result is consistent with that this transient hermaphroditic stage is an essential step in testis development and could not be bypassed. Another alternative explanation is that the transient hermaphrodite stage could be due to sex change in genetic females because there is a tendency of bias toward females in early stages (60−80 dph, **Figure 5C**). However, the deviation from 50:50 was not statistically significant (*P* > 0.05, Fisher’s Exact Test), which could be observed just by chance. Although the sex determination locus was not identified in *L*. *polyactis* and we still cannot rule out an environmental sex determination mechanism (47), we observed sex differences in gonad weight measurements from 50 dph, and ovary weight is significantly higher than testis (**Supplemental Figure 1**). Therefore, it is extremely unlikely that the gonad samples with the transient hermaphrodite stage are genetic females.

### A Transitional Model Between Gonochorism and Hermaphroditism

In this study, *L*. *polyactis*’ unique pattern of testis differentiation is neither typical hermaphrodite nor gonochorism. Because hermaphroditism is extremely rare in the *Sciaenidae* family and its closely related species, *L*. *crocea*, is a differentiated gonochorist with XX/XY sex determination, we speculate that this gonadal differentiation pattern in *L*. *polyactis* evolved from a gonochoristic ancestor. The underlying molecular mechanisms warrant further study. It may serve as a transitional type from gonochorism to hermaphroditism. The male-specific nature of this transient stage is also fascinating, which is different from the zebrafish gonadal development process, in which all individuals of zebrafish need to experience the juvenile ovary stage, with no sex differences in morphology and histology (5, 25, 26).

In addition, sexual dimorphism in germ cell numbers began to appear at 43 dph, and the number of male germ cells is significantly smaller than female germ cells in *L*. *polyactis*. In medaka, a typical gonochoristic fish, the number of germ cells in XX individuals is significantly higher than that of XY fish in the undifferentiated gonads, indicating that sexually dimorphic proliferation is critical for the fate of gonadal sex differentiation (48, 49). Furthermore, in most gonochoristic fishes, sexually dimorphic germ cell proliferation early in development will also affect the final outcome of differentiated sex (50). Depletion of germ cells in zebrafish larvae (51, 52), or reductions of germ cells at the embryonic (53), juvenile (54), or adult stages (54, 55) can all cause female-to-male sex reversal. Therefore, germ cell number is vital to gonad development at all times, from embryos to adults (56). In *L*. *polyactis*, although EPOs are produced at the beginning of the transient hermaphroditic stage in male gonads at 43 dph, the total number of germ cells is significantly lower than that of the ovaries until the end of this stage. This result suggests that differences in germ cell numbers are also important for early-stage gonadal differentiation in *L*. *polyatcis*, similar to gonochoristic fish described above.

Apoptosis of EPOs at this transient hermaphroditic stage in the testis is also an important characteristic of the transitional model. Complete female-to-male sex reversal in zebrafish has been observed in *fancl* mutants when meiotic oocytes undergo apoptosis (57). To our surprise, apoptosis in the testis occurs not only in the germ cells but also in somatic cells. The germ cell number reduction through EPO degeneration in testes may play an essential role in promoting the differentiation and function.

*L. polyactis* has many advantages for gonadal development and differentiation study. The size of the fish is sufficient for tissue and blood sample collection during critical stages of gonad development. A closely related, genome sequenced sister species *L. crocea* is available for comparative genomic studies. These characteristics make the *L*. *polyactis* a natural model for researching the evolution from gonochorism to hermaphroditism in teleost.

### Elevated E2 Level in Males Demarcates the Transient Hermaphroditic Stage in Testis Differentiation

As a natural inducer of ovarian differentiation, estrogen is considered as a key factor in sex differentiation (27, 58). In tilapia, Cyp19a1a expressed as early as 5 dph in XX female fish to drives the differentiation of the ovary (8). In zebrafish, estrogen E2 or bisphenol A (BPA) primarily binds Esr2a to inhibit the expression and action of *dmrt1*, which is a key gene in male sex determination (59). In fact, knockout of male pathway genes or overexpression of female pathway genes often result in the upregulation of *cyp19a1a* and increase estrogen level, promoting ovary development. The opposite is also true (29).

Interestingly, the effect of exogenous estrogen or estrogen-synthesis inhibitors on the treatment of gonochorists and hermaphroditic fish is different. For gonochoristic teleost, treatment of exogenous E2 can reverse genetic males to phenotypic females if the treatment is applied before or during the sex differentiation window (60–62). On the contrary, AI (aromatase inhibitor) treatment of genetic females can induce phenotypic males when applied during the critical period of sex differentiation in a number of fish species (63–65). After treatment, physiological sex is stable with reproductive functions. However, for hermaphroditic teleost, sex reversal induced by E2 and AI is shown to be transient. For estrogen-induced feminization in protandrous black porgy (*Acanthopagrus schlegelii*) (34, 35) and AI-induced masculinization in the protogynous orange-spotted grouper (*Epinephelus coioides*) (66, 67), the original sex is restored after the chemical treatment is withdrawn. The *L. polyactis*’ hermaphrodite stage we discovered is also temporary. In this stage, male serum E2 level peaks at 60 dph, which is significantly higher than developing ovaries. Meanwhile, the androgen (11-KT and testosterone) levels are extremely low. When E2 level drops to an extremely low level from 70 to 90 dph, and the EPOs gradually degenerated and eventually disappeared according to decreasing estrogen level, which resembles the hermaphrodite type.

When the significant elevation of E2 happens between 50 and 60 dph in males, no obvious histological changes are observed. The lack of an immediate increase in oocyte production could be due to a delayed effect of estrogen (59, 68), or the endogenous E2 level at the beginning of this stage is not sufficient to maintain the continued development of oocytes.

Although we discovered E2 level correlates with the transient hermaphroditic stage in testis development and we propose this might be the molecular mechanism of EPO degeneration, we cannot exclude the possibility that other well-characterized sex differentiation factors, such as doublesex, mab-3 related transcription factor 1 (*dmrt1*) (68), anti-Müllerian hormone (*amh*) (7) or germline *α* (*figlα*) (69) et al. are potentially involved in EPOs production and apoptosis.

### 11-KT, Not T, Is the Major Androgens in *Larimichthys polyactis*

In mammals, testosterone (T) is the major androgens and plays a key role in the development of male reproductive tissue such as testis, as well as promoting secondary sexual characteristics such as increased muscle and bone mass and the growth of body hair (70). 11-KT has a similar potency to testosterone as an androgen, and it has been identified as an important adrenal androgen in mammals (71). In most teleost fish, 11-KT is considered as the major endogenous androgenic sex hormone (72). 11-KT is found in higher levels in the plasma of males than in females, whereas this is usually not the case for testosterone. 11-KT is generally more effective than testosterone in stimulating secondary sexual characters, reproductive behavior and spermatogenesis (73).

In *L. polyactis*, we found no significant differences in testosterone levels between males and females during the spawning cycle. However, the 11-KT level demonstrated significant sexual dimorphism at 270–360 dph. The elevation and decrease of the 11-KT level correspond to sperm maturation and gonads degeneration after reproduction, respectively. Although we cannot rule out the role of testosterone in spermatogenesis and maturation, 11-KT is apparently the major androgen regulating spermatogenesis in *L*. *polyactis*. However, the increase of 11-KT is later in gonadal differentiation suggests that this androgen may be not necessary to the testis development, such as in medaka *Oryzias latipes* (74).

### Evolutionary Implications of the Transient Hermaphroditic Stage in Male Testis Differentiation

Regarding fitness, this transient hermaphroditic stage appears to have an evolutionary disadvantage in reproduction by wasting energy through a laborious transient appearance of oocytes followed by immediate apoptosis in testis. Based on the evolutionary relationship among closely related species, we concluded that this type evolved from a gonochoristic ancestor after *L*. *polyactis*’s divergence from its sister species *L*. *crocea.* How did this type of development procedure get fixed in the entire population remains an evolutionary puzzle.

In plants, gonochorism may evolve from hermaphrodites either by gradually increasing in sex-specific investment or occurrence of male- or female-sterility mutations (3). The opposite pattern can also exist because sexual reproduction and sex determination could be lost and re-evolved many times. For example, monoecious evolved from dioecious seven times independently during evolution in the balsam pear family of Cucurbitaceae (75). In fish, the direction of evolution between hermaphrodites and gonochorism, and whether there are intermediate species remained unclear. Based on the observation from its closely related species, *L. polyactis*’ case could be due to selection on recent mutation(s), coping with the population’s sex imbalance for survival under high fishing pressures. Theoretically, the two evolutionary endpoints on a reproductive spectrum are gonochorism and simultaneous hermaphroditism. A wide variety of intermediate mixed-sex modalities can exist, such as gynodioecy (a population mixture of females and hermaphrodites), androdioecy (a mixture of males and hermaphrodites), and trioecy (males, females, and hermaphrodites) (76). Based on the observation of a high male ratio in field *L. polyactis* samples, the androdioecy in the wild population of *L. polyactis* cannot be ruled out. Whether the sexual reproduction type *L. polyactis* will change from gonochorism to complete hermaphroditism is still an open question.

The appearance of the transient hermaphroditic stage in males may be due to a recent response to rapid selection for sexual precocity. Under the stress of overfishing, seawater pollution, as well as ocean current and water quality changes, *L. polyactis’* population become sexual precocity, gender imbalance and body size miniaturization (37, 38, 41). From an evolutionary perspective, being able to reproduce at an early age with a smaller body size will be adaptive for survival under the new selective pressures. Additionally, in the current *L. polyactis* population, although there was no difference in body size between males and females during breeding, compared to slimmer males, females have enlarged belly during pregnancy, which makes them less like to escape. This may explain the higher proportion of males caught in the wild during the non-reproductive season.

Taken together, our findings support a new model for sex differentiation form in *L. polyactis*. According to this model, a transient hermaphroditic stage was contributed by the absence of androgens (11-KT) and elevated E2 levels. Meanwhile, testicular oocytes in the hermaphroditic stage degenerated by apoptosis but were rescued and maintained by exogenous E2. Our results strongly indicate that male gonad differentiation in *L. polyactis* is a special case, presumably due to mutations in the sex determination and differentiation pathway. This alteration in sexual reproduction type could be driven by the positive selection for smaller body size and sexual precocity due to the fishing pressure, and it may lead to the eventual transition from gonochorism to hermaphroditism. Our study provided an interesting model for understanding the evolution of hermaphroditism and gonochorism. The key genes and underlying molecular mechanisms warrant further investigation.

## Data Availability Statement

The raw data supporting the conclusions of this article will be made available by the authors, without undue reservation.

## Ethics Statement

The animal study was reviewed and approved by Committee of Laboratory Animal Experimentation at Zhejiang Academy of Agricultural Sciences.

## Author Contributions

Q-PX and BL conceived and designed the experiments. B-BL, WZ, FL, and PT collected sample. Q-PX, B-BL, and PT performed the experiments. Q-PX and B-BL performed the analyses. Q-PX, XW, and BL wrote the paper. All authors contributed to the article and approved the submitted version.

## Funding

This work was supported by the Natural Science Foundation of Zhejiang Province (grant number LQ19C190002), the National Natural Science Foundation of China (grant number 31902341), The Key National and Special Project of Blue Granary Science and Technology Innovation (grant number 2018YFD0901204), the Science and Technology Department of Zhejiang Province (grant number 2017C02013), and the Science and Technology Department of Xiangshan county (grant number 2019C0001). XW is supported by the USDA National Institute of Food and Agriculture (Hatch project 1018100), National Science Foundation EPSCoR RII Track-4 Research Fellowship (NSF OIA 1928770), an Alabama Agricultural Experiment Station Enabling Grant, as well as a generous laboratory start-up fund from Auburn University College of Veterinary Medicine.

## Conflict of Interest

The authors declare that the research was conducted in the absence of any commercial or financial relationships that could be construed as a potential conflict of interest.

## Abbreviations

*L. polyactis*, *Larimichthys polyactis*; dph, days post hatching; H.E., hematoxylin–eosin; EGSCs, embryonic germ stem cells; SO, somatic cell; OSCs, oogonial stem cells; EPOs, early primary oocytes; OC, oocytes; SSCs, spermatogonia stem cells; SC, spermatocytes; SP, sperm; SD, spermatid; GSI, gonadosomatic index; E2, 17*β*-estradiol; 11-KT, 11-ketotestosterone; dpf, days post fertilization.
