# Supplementary material for: A Transient Hermaphroditic Stage in Early Male Gonadal Development in Little Yellow Croaker, Larimichthys polyactis
Source: Front Endocrinol (Lausanne). 2021 Jan 27;11:542942. doi: 10.3389/fendo.2020.542942 (PMC7873647; doi:10.3389/fendo.2020.542942)
Supplement: Supplementary file 1 [file Image_1.pdf]

Supplemental Figure 1

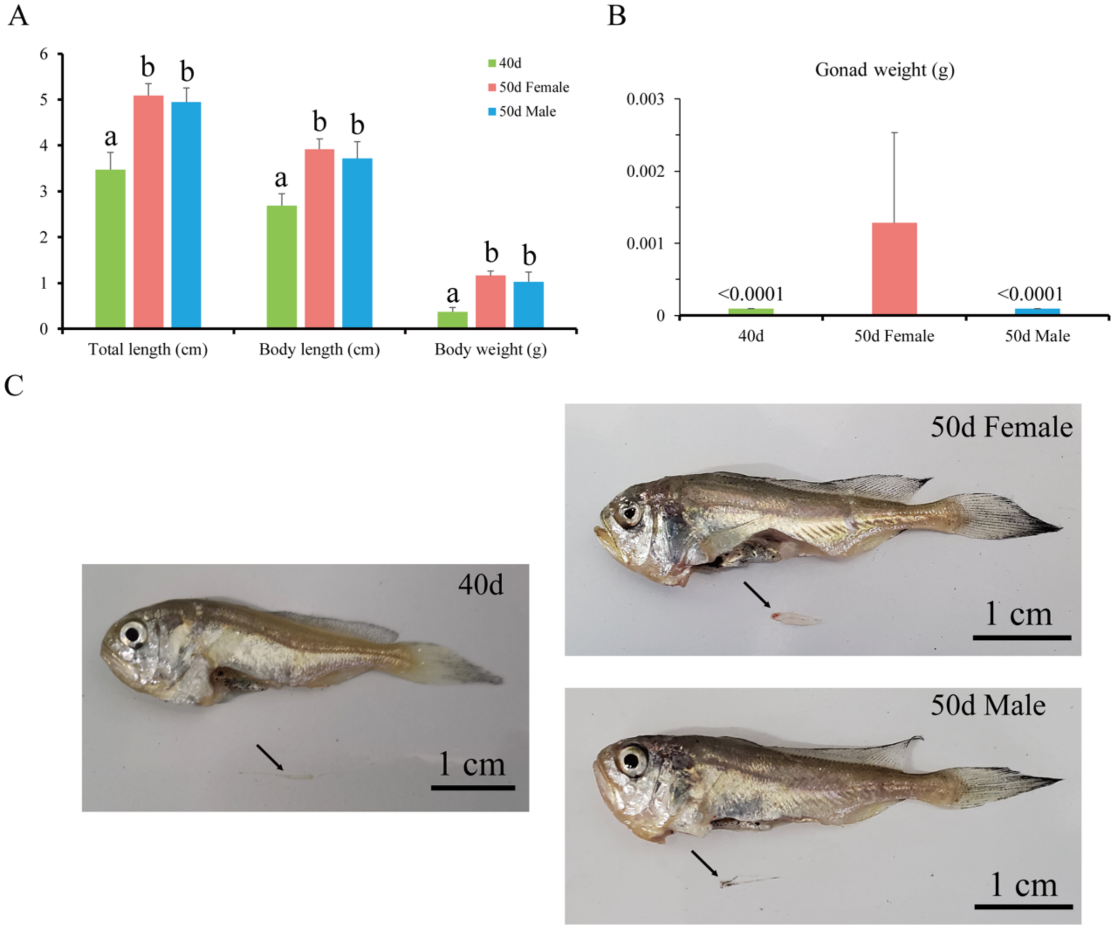

Supplemental Figure legend

Supplemental Figure 1. Comparison of body length, body weight, gonad weight and size at 40 and 50 dph in *L. polyactis*.

(A) The total length, body length and body weight of *L. polyactis* were measured and compared in indistinguishable sex at 40 dph, females and males at 50 dph. Different lowercase letters above the error bar indicate statistical differences between indistinguishable sex 40 dph, females and males at 50 dph, at $P < 0.05$ as determined by one-way ANOVA followed by Duncan's post hoc test. (B) The gonadal weight was measured and compared in indistinguishable sex at 40 dph, females and males at 50 dph. (C) Anatomical examination of the gonads in the indistinguishable sex at 40 dph, females and males 50 dpf.
